# Using an NMR metabolomics approach to investigate the pathogenicity of amyloid-beta and alpha-synuclein

**DOI:** 10.1007/s11306-017-1289-5

**Published:** 2017-10-29

**Authors:** M. M. Phelan, E. Caamaño-Gutiérrez, M. S. Gant, R. X. Grosman, J. Madine

**Affiliations:** Institute of Integrative Biology, University of Liverpool, Liverpool, UK

**Keywords:** Metabolomics, Parkinson’s disease, Alzheimer’s disease, Alpha-synuclein, Amyloid-beta, NMR spectroscopy

## Abstract

**Introduction:**

The pathogenicity at differing points along the aggregation pathway of many fibril-forming proteins associated with neurodegenerative diseases is unclear. Understanding the effect of different aggregation states of these proteins on cellular processes is essential to enhance understanding of diseases and provide future options for diagnosis and therapeutic intervention.

**Objectives:**

To establish a robust method to probe the metabolic changes of neuronal cells and use it to monitor cellular response to challenge with three amyloidogenic proteins associated with neurodegenerative diseases in different aggregation states.

**Method:**

Neuroblastoma SH-SY5Y cells were employed to design a robust routine system to perform a statistically rigorous NMR metabolomics study into cellular effects of sub-toxic levels of alpha-synuclein, amyloid-beta 40 and amyloid-beta 42 in monomeric, oligomeric and fibrillar conformations.

**Results:**

This investigation developed a rigorous model to monitor intracellular metabolic profiles of neuronal cells through combination of existing methods. This model revealed eight key metabolites that are altered when neuroblastoma cells are challenged with proteins in different aggregation states. Metabolic pathways associated with lipid metabolism, neurotransmission and adaptation to oxidative stress and inflammation are the predominant contributors to the cellular variance and intracellular metabolite levels. The observed metabolite changes for monomer and oligomer challenge may represent cellular effort to counteract the pathogenicity of the challenge, whereas fibrillar challenge is indicative of system shutdown. This implies that although markers of stress are more prevalent under oligomeric challenge the fibrillar response suggests a more toxic environment.

**Conclusion:**

This approach is applicable to any cell type that can be cultured in a laboratory (primary or cell line) as a method of investigating how protein challenge affects signalling pathways, providing additional understanding as to the role of protein aggregation in neurodegenerative disease initiation and progression.

**Electronic supplementary material:**

The online version of this article (doi:10.1007/s11306-017-1289-5) contains supplementary material, which is available to authorized users.

## Introduction

Neurodegenerative diseases are a burden worldwide, ever increasing as the population ages. Three of the most common neurodegenerative diseases Alzheimer’s disease (AD), Dementia with Lewy bodies (DLB) and Parkinson’s disease (PD) are associated with misfolded protein aggregates. AD is characterised by extracellular plaques containing amyloid-beta (Aβ) (Selkoe 1991). DLB and PD have intracellular inclusions called Lewy bodies (LBs), containing α-synuclein (asyn) (Spillantini et al. 1997). Aβ and asyn can misfold and aggregate into amyloid-like filamentous assemblies. Self-assembly proceeds *via* one or more partially folded intermediates, or oligomers, which can propagate in various ways to form either the insoluble fibrils or precipitate as amorphous aggregates. The exact nature of the pathogenic amyloid species is a matter of intense debate, but there is increasing evidence that prefibrillar oligomers or intermediates play a role in pathogenicity (Haass and Selkoe 2007). There is a need to address the nature of these effects of the different levels of conformational complexity that these proteins can adopt during aggregation and disease progression.

AD is associated with fibrillar deposits of the 39–42 amino acid protein Aβ arising from enzymatic cleavage of the extracellular portion of the transmembrane amyloid-precursor protein. The two most abundant forms are Aβ40 and Aβ42, which are 40 and 42 residues in length, respectively. Aβ40 is the most common form encompassing 80–90% of total Aβ (Murphy et al. 2010), but it is suggested to be less toxic, and therefore less involved in protein pathogenicity (Klein et al. 1999). Aβ42 only represents 5–10% of total Aβ, but it has been identified as the major component of plaques in the brain (Selkoe 2001) and shown to aggregate faster than Aβ40 (Jarrett et al. 1993). It is hypothesised that the Aβ42/40 ratio may be the dictating factor in the pathogenic effects of Aβ (Wolfe 2007).

Asyn is a native protein found in neuronal synapses concentrated at the presynaptic termini (Iwai et al. 1995). Its native function is unknown, although research suggests a role in synaptic vesicle maintenance through association with cellular membranes (Outeiro and Lindquist 2003) and a role in dopamine regulation (Perez et al. 2002). LBs are formed in the neurons in PD and DLB and are primarily composed of asyn, along with other proteins and lipids (Goldberg and Lansbury 2000).

The aggregation pathways of Aβ and asyn are central to neurodegenerative disease research into diagnostics and therapeutic targets due to mounting evidence that soluble forms of the proteins are more toxic than insoluble fibrils. However, the mechanism by which these proteins cause cell apoptosis remains unknown. Animal models capture some of the disease characteristics yet do not cover the range of responses found in humans. Here we utilise an existing cellular model (Xicoy et al. 2017) and adapt metabolomic fingerprinting techniques (Beckonert et al. 2007) to establish a robust model for probing intracellular metabolic profiles of neuronal cells. We employ nuclear magnetic resonance (NMR) metabolomics to identify some of the mechanisms involved in the cellular disruptions that Aβ40, Aβ42 and asyn in their different conformations cause using a cellular model. Extracellular protein challenge was selected to reflect the body of mounting evidence suggesting that cell-to-cell transmission occurs extracellularly or within vesicles highlighting the importance of understanding the effect of external protein challenge on cellular mechanisms (Lee et al. 2010). This investigation revealed eight key metabolites that are altered when neuroblastoma cells are challenged with Aβ and asyn in different aggregation states. Pathway analysis and known functions of these metabolites confirmed that a range of metabolic processes and mitochondrial function are altered within cells in response to these proteins. This work provides a model system with relevance to AD, DLB and PD initiation and progression with potential for future development to enhance understanding of the role of protein aggregation in neurodegenerative diseases. In addition, this model could assist future clinical applications in diagnostics through biomarker identification, early detection systems and preliminary therapeutic testing.

## Experimental

### Protein expression and preparation

Asyn and Aβ were expressed in *E. coli* BL21 cells according to established protocols (Finder et al. 2010; Madine et al. 2009). Asyn oligomers were produced by dissolving lyophilised protein in 50 mM sodium phosphate buffer, pH 7.0, containing 20% ethanol to a final concentration of 7 μM and shaking for 4 h. Oligomers were lyophilised and resuspended in 50% of the starting volume of 50 mM sodium phosphate buffer, pH 7.0, 10% ethanol and stirred with open lids for 24 h at room temperature (Danzer et al. 2007). Asyn fibrils were formed by incubation at 20 μM in 10 mM Tris, pH 7.4, with agitation at 37 °C for 2 weeks (Madine et al. 2009).

Aβ40 oligomers were produced by incubating Aβ40 at 2.5 mg/mL in hexafluoro-2-propanol (HFIP) for 15 min at 25 °C and then diluting 10× in double distilled water (ddH_2_O) with incubation for another 15 min at 25 °C prior to lyophilisation (Haupt et al. 2012). Aβ42 oligomers were produced following dissolution at 1 mM in HFIP and evaporation under vacuum, followed by resuspension to 5 mM in dimethylsulfoxide and dilution to 100 μM in Ham’s F-12 with 24 h incubation at 4 °C (Doi et al. 2009). Aβ fibrils were formed by incubation at 20 μM in 50 mM sodium phosphate, 100 mM sodium chloride, pH 7.4, with agitation at 37 °C for 2 weeks (Madine et al. 2009). Proteins were added to cells at 5 μM following dilution in cell culture medium. Transmission electron microscopy was used to confirm protein species (see Supplementary Fig. 1 in Supplementary Material).

### Cell culture and protein challenge

SH-SY5Y cells (European Collection of Cell Cultures, ECACC) were exposed to each sample condition; control (no protein added), Aβ40, Aβ42 and asyn (monomer, oligomer and fibril) in T25 flasks at ~80% confluence and incubated for 24 h prior to harvest *via* trypsinisation. Samples were centrifuged for 3 min at 300 g, media was discarded and cells were washed twice in 1 mL phosphate-buffered saline and centrifuged again. Cell pellets were stored at − 80 °C. All conditions were tested in three biological replicates, and each biological replicate provided two technical replicates of ~150,000 cells per sample. Cells were assessed for viability prior to freezing using trypan blue (Strober 2001).

### Metabolite extraction

Five hundred microliter of 50:50v/v ice cold acetonitrile:H_2_O was added to cell pellets and incubated on ice for 10 min. Samples were sonicated in an ice bath for three 30 s bursts (with 30 s rest) at 23 kHz and 10 µm amplitude. Sonicated samples were centrifuged at 12,000 g for 5 min at 4 °C and the supernatant lyophilized (Beckonert et al. 2007).

### Sample preparation for NMR

Each sample was resuspended in 200 µL of deuterated buffer (100 mM sodium phosphate buffer pH 7.4, with 100 µM d4 trimethylsilyl propionate (TSP) and 0.05% NaN_3_), vortexed for 20 s and centrifuged at 12,000 g for 1 min at 20 °C. One hundred and eighty microliter of each sample was transferred to 3 mm (outer diameter) NMR tubes for acquisition.

### NMR acquisition and spectral processing

1D Nuclear Overhauser Effect (1D NOE) and Carr-Purcell-Meiboom-Gill (1D CPMG) NMR spectra were acquired at 600.13 MHz using a Bruker Avance III 600 MHz NMR spectrometer fitted with a 5 mm TCI cryoprobe. Samples were referenced to TSP at 0 ppm. 1DNOEs had spectral widths of 30 ppm, 4 s relaxation delay, 32 scans collected with 96k data points (2.727 s acquisition time). 1D CPMGs had spectral widths of 20 ppm, 4 s relaxation delay, 256 scans with 32 dummy scans collected into 72k data points (3.067 s acquisition time). Temperature was calibrated to 25 °C (± 0.1 °C) via methanol thermometer (Ammann et al. 1982) prior to acquisition and receiver gain was constant for all acquisition. All spectra were zero filled to 128k data points with exponential line broadening of 0.3 Hz applied before Fourier transformation, spectra were automatically phased, referenced and baseline corrected in Topspin 3.1 (Bruker, UK).

### Metabolite annotation and identification

Metabolites in the cell extract 1D CPMGs spectra were annotated using Chenomx 8.2 (Chenomx, Canada) and subsequently identified where indicated using an in-house library. Spectra were subjected to a quality control filter prior to statistical analysis by characterization of the TSP reference peak with spectra excluded that did not satisfy linewidth or baseline requirements in the spectral bucketing software AMIX (Bruker, UK). Spectra were prepared for statistical analysis by normalizing each spectrum using the probabilistic quotient normalisation (PQN) method (Kohl et al. 2012) and spectra bucketed according to spectral features or peaks; all peaks both annotated and unknown were included in the bucket table. All spectra are available via Metabolights (MTBLS455) with acquisition and processing parameters.

### Statistical analyses

Buckets corresponding to unidentified metabolite peaks were removed from the bucket table. The resultant data was mean centered and scaled by standard deviation prior to linear discriminant analysis of principal component using the ‘DAPC’ function in the ‘adegenet’ package (version 1.4-2) (Jombart 2008) in order to create two models: (i) conformation model (four groups): control, monomer, oligomer and fibril; (ii) combined model (ten groups): control, Aβ40 monomer, Aβ40 oligomer, Aβ40 fibril, Aβ42 monomer, Aβ40 oligomer, Aβ40 fibril, asyn monomer, asyn oligomer and asyn fibril. Cross-validation was performed to identify the number of principal components to retain the linear discriminant analysis (LDA) model. Details of cross validation are available with deposited data (Metabolights MTBLS455). The conformation model was built using metabolite annotated buckets only and comprised 14 principal components and three linear discriminants and for the combined model 12 principal components and three linear discriminants were retained. Both score plots of the models and most contributing variables are reported. Other statistical analyses and graphical representations of the models were performed in the statistical packages within the software R, (Team R, Cranfield, UK) (Febrero-Bande et al. 2012).

### Biological contextualisation

Analysis was carried out using the most influential metabolites (top 10% from loadings; see Supplementary Table 1 in Supplementary Material) of DAPC models with KEGG (Ogata et al. 1999), and MetaboAnalyst (Xia et al. 2002). All the Human pathways in the KEGG database were used to collate a library of compound associated pathways. The matching pathways were then tabulated and ranked according to pathway type and within each type to number of metabolites represented in the pathway. Pathway analysis was compared to the automated pathway analysis available in the MetaboAnalyst online server the results of which were found to be comparable to KEGG analysis. Metabolites set enrichment analysis (MSEA) was performed using an EASE score modified Fisher exact test as implemented in DAVID (Huang da et al. 2009). The metabolite set was created by downloading all *Homo sapiens* related pathways of KEGG database version 070417 (Ogata et al. 1999) resulting in a total of 5432 metabolites represented in 250 pathways. The 26 metabolites identified as contributing to the separation of both DAPC models and reported in Supplementary Table 1 were used for enrichment. All p-values were corrected for false discovery rate by Benjamini–Hochberg.

## Results

### Spectral observations

**Fig. 1:**
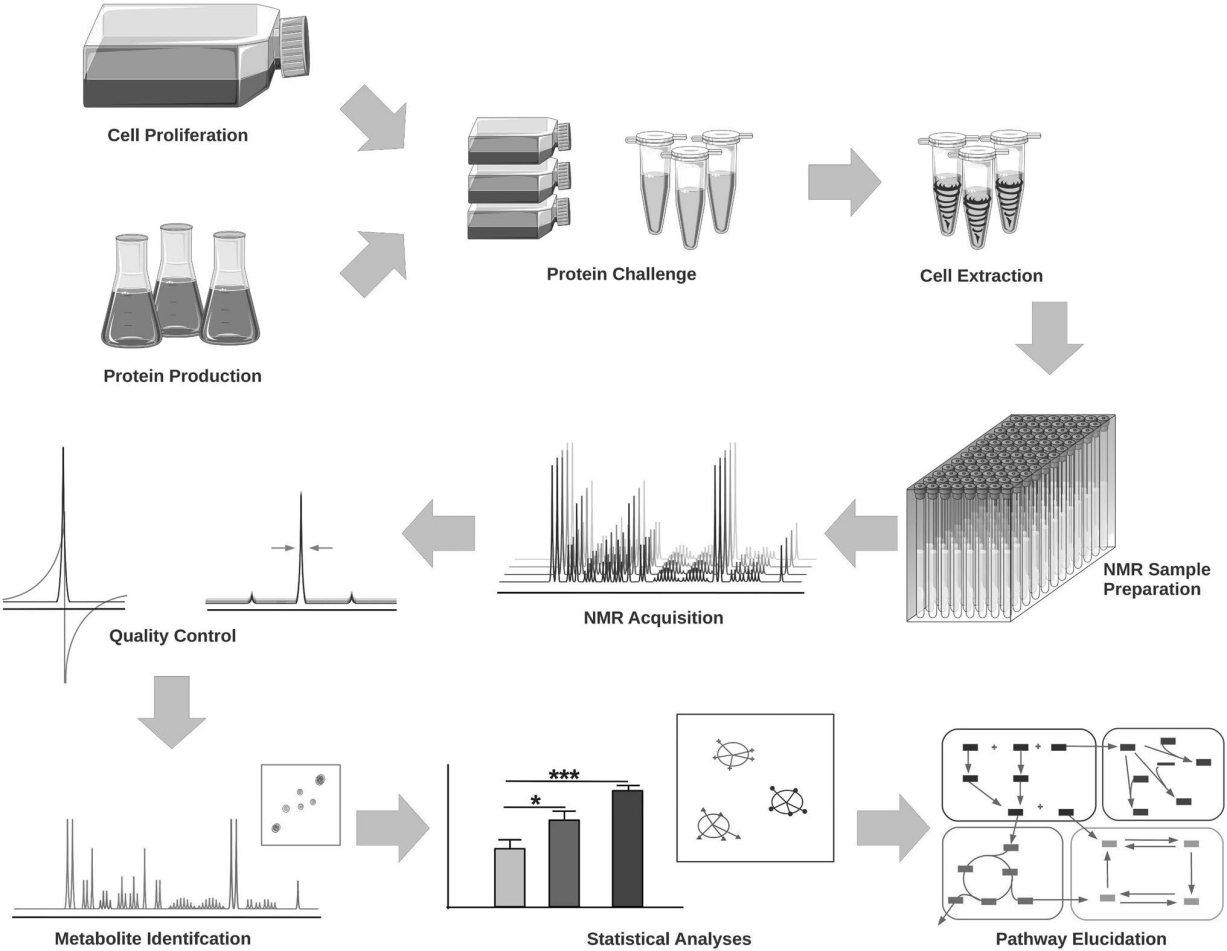
Workflow showing experimental and analysis pipeline. Proteins (Aβ40, 42 and asyn) were recombinantly expressed in *E. coli* and used as freshly prepared (monomer) or following incubation to form oligomers or fibrils using established protocols (see methods). SH-SY5Y neuroblastoma cells were grown until sufficient cells for three biological and two technical repeats per condition were available (~150,000 cells per sample). Cells were challenged with protein samples (5 µM) for 24 h prior to harvest and extraction. NMR samples were prepared and spectra acquired with normalisation to TSP as an internal standard for quality control. Metabolites were identified and confirmed using Chenomx and an in-house metabolite library. Statistical analysis and cross-validation were carried out using linear discriminant analysis, with KEGG and MetaboAnalyst employed for pathway inference

In order to effectively monitor metabolite levels in neuronal cells the workflow designed for this project was carefully considered and optimised (Fig. 1). The SH-SY5Y cells were exposed to sub-toxic levels of either, predominantly monomers (freshly prepared), oligomers or fibrils of three proteins involved in neurodegenerative diseases (Aβ40, Aβ42 and asyn) for 24 h at which time live cells (over 90% in all groups) were harvested for metabolite extraction. The metabolite extract spectra displayed a consistent set of metabolite signals present with multiple metabolites identified from 1D multiplet pattern overlap (see Supplementary Fig. 2 in Supplementary Material). The spectra were divided into 232 buckets consisting of either individual peaks or groups of multiplets with 185 (79%) assigned to 78 metabolites.

### Statistical analysis

**Fig. 2:**
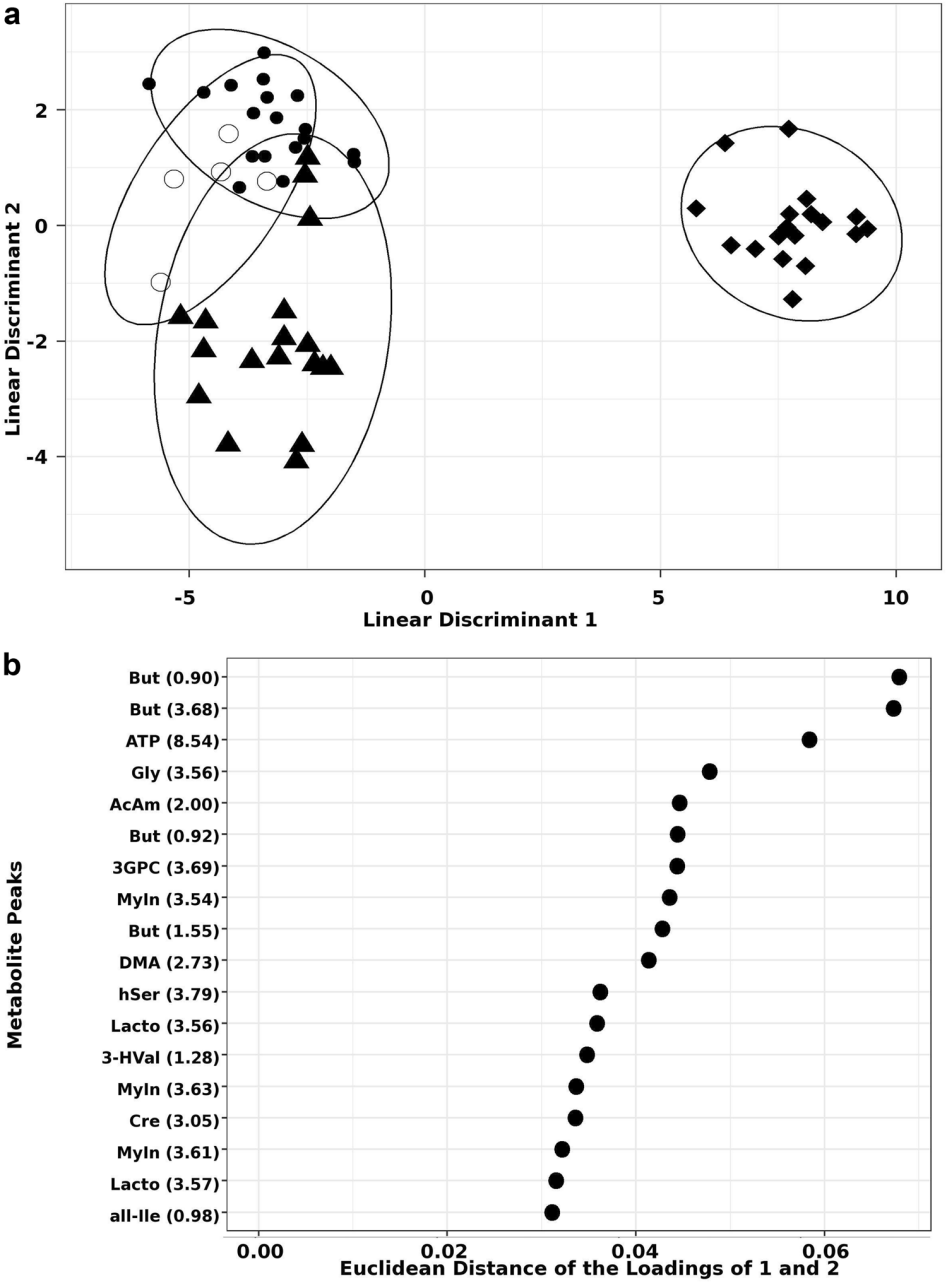
Conformation DAPC model. **a** Conformation based separation of protein challenges; control (open circles), monomer (filled triangles), oligomer (filled circles) and fibril (filled diamonds) represented as the Euclidean distance of the vector formed by the loadings of linear discriminant 1 and 2. Each point is the metabolite profile of one sample and the ellipse represents the 95% region around the mean of the points of each group. **b** Key metabolites identified to contribute to the top 10% variance between the groups with average ppm of peak indicated; butyric acid (But), adenosine triphosphate (ATP), glycine (Gly), acetamide (AcAm), sn-glycero-3-phosphocholine (3GPC), myoinositol (MyIn), dimethylamine (DMA), homoserine (hSer), lactose (Lacto), 3-hydroxyvalerate (3-HVal), creatinine (Cre), alloisoleucine (all-Ile)

The bucketed spectra were subjected to a series of statistical analyses. Multivariate analysis by DAPC was employed to maximise the variation between groups and identify metabolite peaks responsible for groupings. In order to test the cellular response to different protein conformations (monomer, oligomer and fibril), as well as different proteins (asyn, Aβ40 and Aβ42), DAPC was used to create two statistical models from this data; conformation and combined. The conformation DAPC model showed excellent discrimination between protein conformations (Fig. 2a) and was able to correctly re-classify all samples with an accuracy of 93.2%, with fibrils showing greatest separation from control. Individual group reclassification accuracies are reported in Supplementary Table 2. The top 10% buckets that contribute the most to this separation are shown in Fig. 2b, with those contributing to the top 25% shown in Supplementary Fig. 3.

**Fig. 3:**
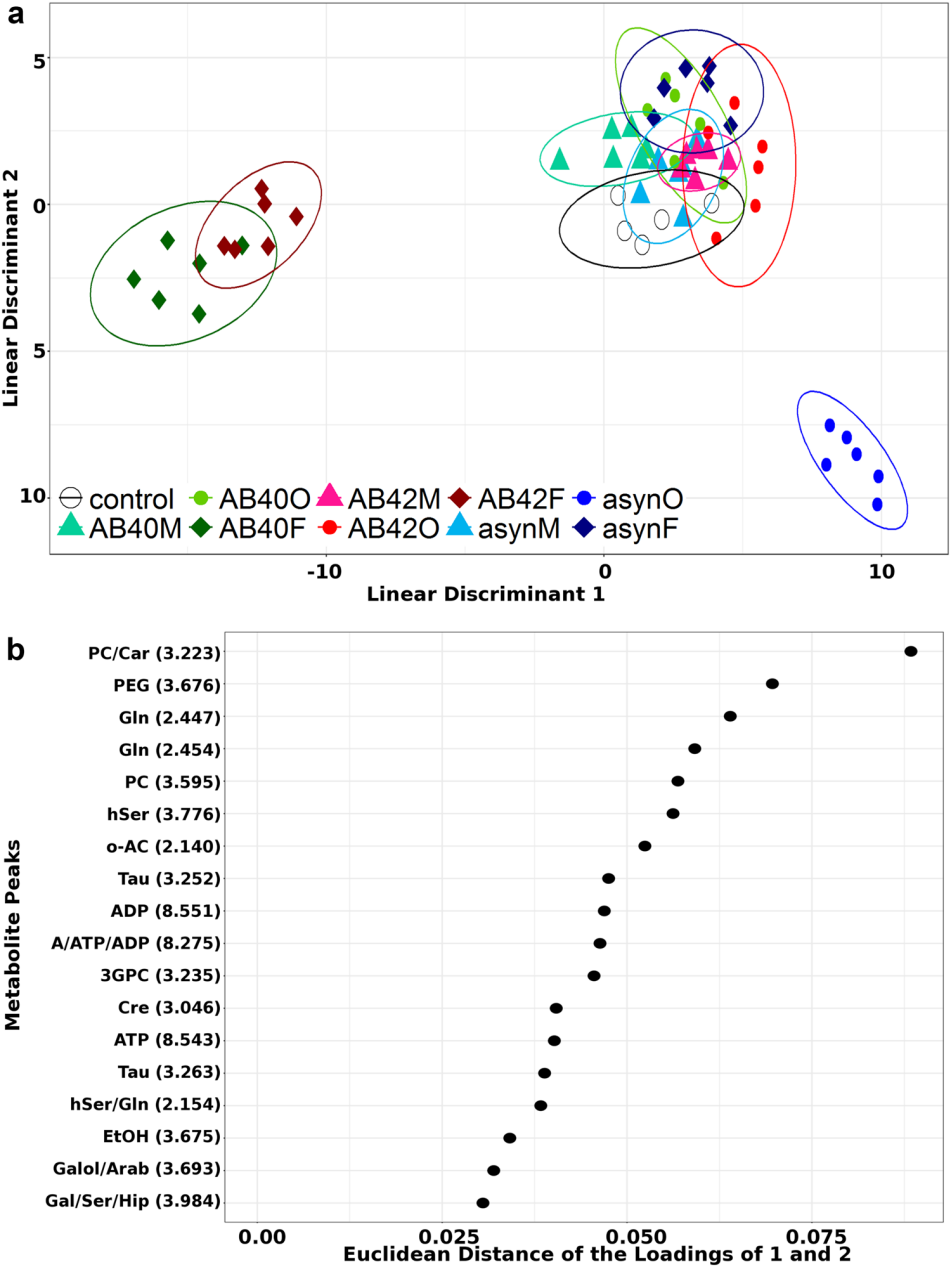
Combined DAPC model. **a** Combined separation of each protein; control (white circles), monomer (M), oligomer (O) and fibrillar (F) challenge for Aβ40 (green shades), Aβ42 (red shades) and asyn (blue shades) represented as the Euclidean distance of the vector formed by the loadings of linear discriminant 1 and 2. Each point is the metabolite profile of one sample and the ellipse represents and the ellipse represents the 95% region around the mean of the points of each group. **b** Key metabolites identified to contribute to the top 10% variance between the groups with average ppm of peak indicated; phosphorylcholine (PC), carnitine (Car), polyethylene glycol (PEG), glutamine (Gln), hSer, acetylcholine (o-Ac), taurine (Tau), adenosine diphosphate (ADP), ATP, adenosine (A), 3GPC, Cre, ethanol (EtOH), galactitol (Galol), arabinose (Arab), galactose (Gal), serine (Ser), hippurate (Hip)

A combined model was employed to further explore the differences between proteins, and was able to correctly re-classify all different samples with an accuracy of 89.8% with group specific accuracies reported in Supplementary Table 2. Plot of the first two linear discriminants is shown in Fig. 3a and further complemented with Supplementary Fig. 5 showing the first and third linear discriminants. Fibrils are once more separated from most of the other groups with Aβ40 and Aβ42 fibrils presenting the largest fingerprinting changes. Interestingly, asyn oligomers presented the largest effect in the metabolite profiles, closely followed by fibrils. Ten percent most contributing metabolites responsible for this separation are shown in Fig. 3b, with those contributing to the top 25% shown in Supplementary Fig. 4.

**Fig. 4:**
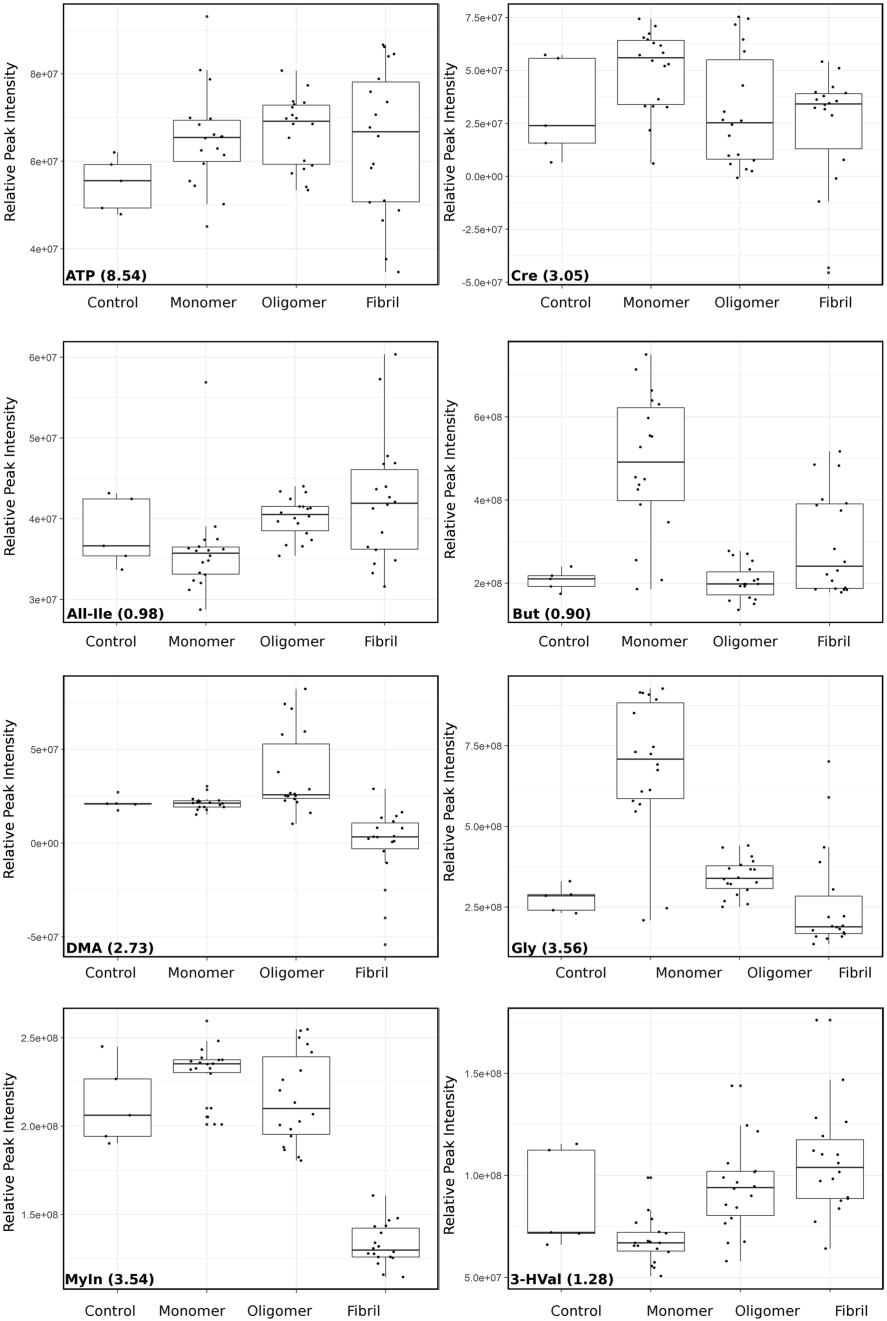
Peak intensity changes for key metabolites from conformation analysis. Boxplots showing change in peak intensity of peaks corresponding to metabolites that contribute to top 10% variance. Where multiple peaks were available for each metabolite a representative peak was selected and ppm value given. Each spectrum within a group is shown as a black square overtop the boxplot (25–75%) with median, and whiskers (5–95%)

**Fig. 5:**
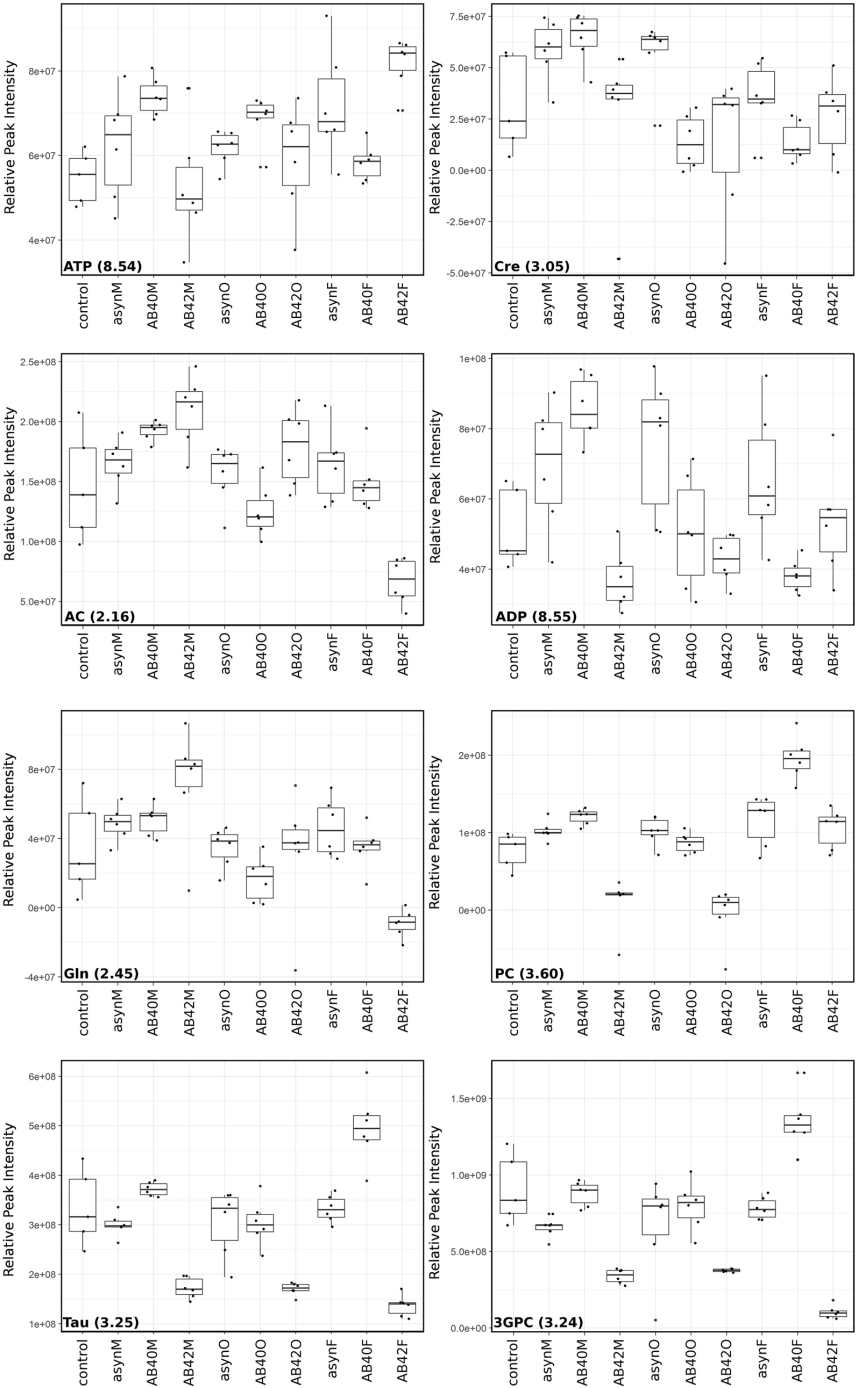
Peak intensities changes for key metabolites from combined analysis. Boxplots showing change in peak intensity of peaks corresponding to metabolites that contribute to top 10% variance. Where multiple peaks were available for each metabolite a representative peak was selected and ppm value given. Each spectrum in the group is shown as a black square overtop the boxplot (25–75%) with median, and whiskers (5–95%)

Correlations were calculated between all buckets and used to confirm confidence in metabolite identification and identify contribution from overlapped peaks (see Supplementary Table 1 in Supplementary Material). These analyses resulted in eight metabolites with high confidence that contribute to the differences for both models. It is noted that most of the peaks that are not observed to contribute to the top 10% in both models do appear in the top 25% in both models (see Supplementary Figs. 3, 4 in Supplementary Material). Three metabolites (butyric acid, glycine, 3-hydroxyvalerate) were identified to contribute only to conformation analysis but were not present in the top 25% for the combined analysis. Conversely acetylcholine is only observed to contribute to combined analysis. Further analysis was carried out on intensity changes of metabolites contributing to conformation (Fig. 4) and combined challenge (Fig. 5). Identified sugars showed low correlation between putative peaks what was attributed to incomplete identification of saccharide metabolites due to overlapping peaks/buckets in the 1D spectra, therefore these were not considered in the biological contextualisation. However it should be noted that sugar metabolism is likely to be altered in neuronal cells in response to protein challenge and should be investigated further in a subsequent study. Similarly, carnitine, adenosine and homoserine were present as overlapping peaks or with low correlation so were omitted from further analysis, along with polyethyleneglycol, acetamide and ethanol which were not thought to be relevant to neuronal cell metabolism.

### Biological contextualization

**Fig. 6:**
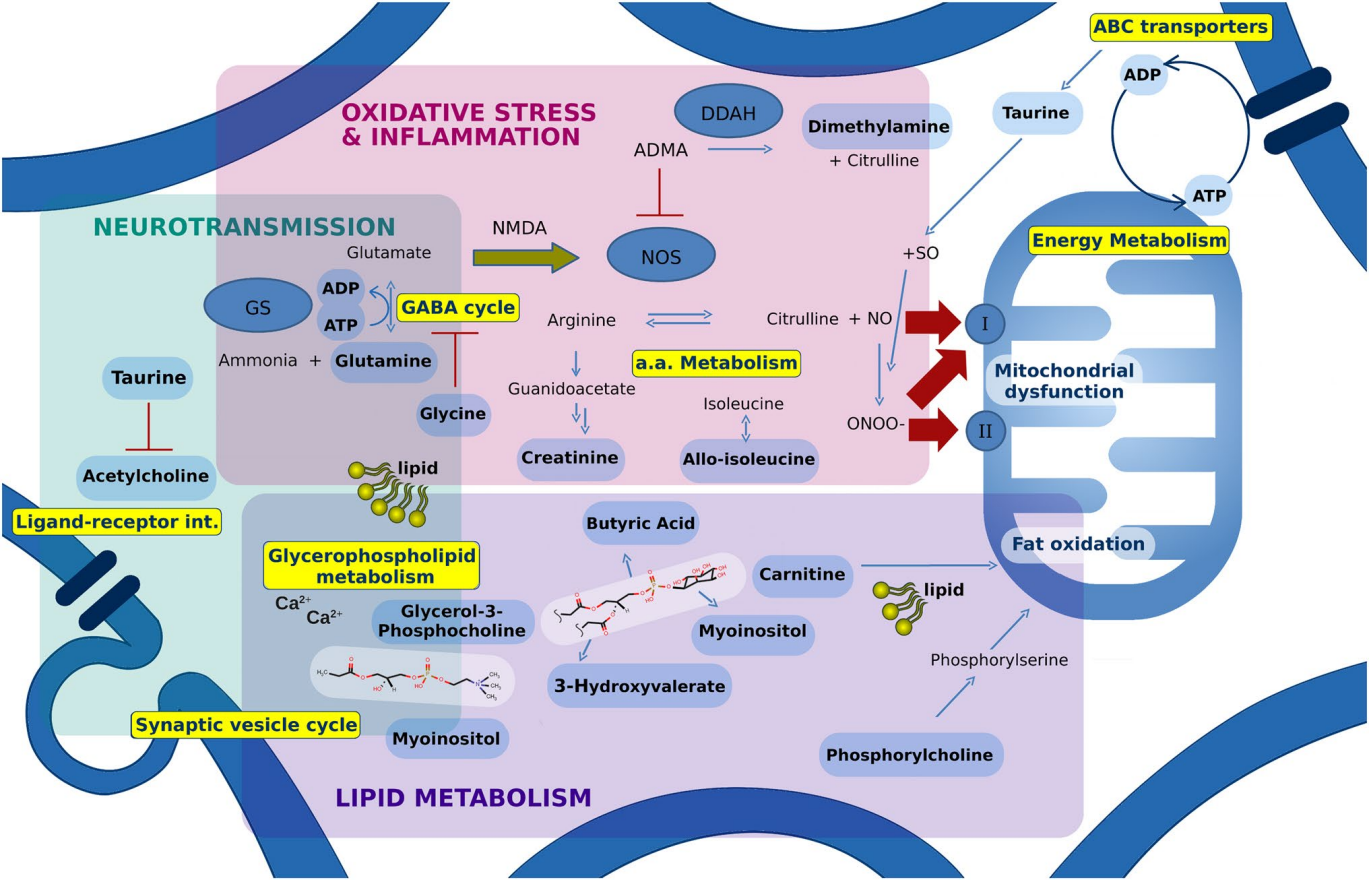
Pathways affected by protein conformational challenge. Figure shows the three main areas identified to be affected; lipid metabolism, oxidative stress and inflammation and neurotransmission. Metabolites identified as significantly varied are shown in bold. Proposed potential pathways including those highlighted by MSEA (see Supplementary Table 4 in Supplementary Material) involved are shown in yellow boxes. Enzymes are shown as blue circles; dimethylarginine dimethylaminohydrolase (DDAH), glutamine synthetase (GS), nitric oxide synthase (NOS). Nitric oxide (NO) can cause mitochondrial dysfunction through damaging mitochondrial complex I directly or *via* reaction with superoxide (SO) to form peroxynitrite (ONOO-), in turn damaging MC I or II

Pathway investigation of key metabolites identified above suggested that protein challenge altered the ability of cells to regulate synthesis of membrane lipid components, amino acids and sugars (see Supplementary Table 3 in Supplementary Material). In addition, many of the metabolites identified are associated with oxidative stress, inflammation and neurotransmission (Fig. 6).

### Lipid synthesis

Phosphorylcholine (PC) is a key component of mitochondrial and cell membranes and is associated with mitochondrial function and the electron transport system (Baykal et al. 2008). It is well established that mitochondrial dysfunction occurs in AD and PD and is therefore likely to be involved in pathology but the exact role remains unclear (Lei and Powers 2013). We observed that sn-3-glycero-phosphocholine (3GPC) and PC were increased in our cells following challenge with asyn oligomers and decreased with fibrils (Fig. 5). Aβ has been shown to induce significant alterations in mitochondrial membrane phospholipids (Rosales-Corral et al. 2012). PC decreases with age and ApoE4 carriers with AD have significantly lower levels of PC consistent with the decrease observed here in the presence of fibrils (Han et al. 2011). PC supplementation has been suggested to improve memory loss in animal studies (Moriyama et al. 1996), and may represent a therapeutic avenue for further exploration in the model developed here. Elevated 3GPC has been reported as an indication for increased phospholipid turnover and membrane defects (Farber et al. 2000). Data presented here suggests that membrane defects may be due to asyn oligomers that increase 3GPC levels (Fig. 4), consistent with reported membrane-penetrating activity of oligomers (Quist et al. 2005).

Consistent with regulation of lipid synthesis the fatty acids 3-hydroxyvaleric acid and butyric acid show altered levels dependent upon protein conformation (Fig. 4). In addition, myoinositol, a structural component of phospholipids and also associated with mitochondrial dysfunction was increased slightly upon challenge with monomers and decreased with fibrils (Fig. 4). Pathway analysis also highlighted myoinositol can function within synaptic vesicle recycling (see Supplementary Tables 3 and 4 in Supplementary Material). Asyn is thought to play a role in maintenance of synaptic vesicle stores with impairment of response to prolonged, low frequency pulse stimulation (Abeliovich et al. 2000). Mice lacking asyn have up to 50% reduction in levels of synaptic vesicles present in the reserve and resting pools (Cabin et al. 2002).

### Oxidative stress, neurotoxicity and inflammation

We observed changes in metabolites associated with regulating response to oxidative stress, neurotoxicity and inflammation (taurine, creatinine, l-alloisoleucine, dimethylamine, glutamine). Taurine can stabilise cell membranes assisting in ion transport and is suggested to have antioxidant properties and to increase cognitive function. Taurine treatment has been successful in recovering cognitive ability in animal models of AD (Kim et al. 2014b). Data presented here shows a decrease in taurine following challenge with fibrils (Figs. 4, 5), consistent with fibrils causing damage to cells through loss of control of membrane transport and oxidative regulation.

An increase in creatinine was observed in response to monomers and Aβ40 oligomers (Figs. 4, 5). In a previous metabolomics study creatinine levels were observed to increase in transgenic AD mice prior to the observance of amyloid plaques consistent with pre-fibrillar species causing metabolic disruption (Lalande et al. 2014). Creatinine supplementation has anti-oxidant properties and protects against cognitive decline and mitochondrial dysfunction in mice models of neurodegenerative diseases (Bender et al. 2008; Klopstock et al. 2011).

Other metabolites (dimethylamine and glutamine) with altered levels during this study are also associated with oxidative and nitrative stress conditions. Dimethylamine showed an increase when challenged with oligomers, with a decrease observed in the presence of fibrils (Fig. 4). Dimethylamine has a downstream role affecting mitochondrial function through generation of Nitric oxide (NO) by nitric oxide synthase (NOS). NO reacts with superoxide to form peroxynitrite which can induce oxidative/nitrative stress conditions and in turn damage to mitochondrial complex I, complex II, and mitochondrial aconitase. NO itself can also damage mitochondrial complex I. NO synthesis by NOS from arginine can be competitively blocked by endogenous inhibitors such as asymmetric dimethylarginine (ADMA) (Fig. 6). ADMA is degraded by dimethylarginine dimethylaminohydrolase (DDAH) to dimethylamine and citrulline. NO synthesis can be stimulated by glutamate *via* the *N*-methyl d-aspartate (NMDA) receptor. Glutamine is the precursor for glutamate generated through the glutamate and γ-aminobutyric acid (GABA) cycle (Fig. 6). An increase in glutamine was reported in the CSF of PD patients in a previous study suggesting a role for GABA cycle regulation of glutamate-glutamine levels in PD (Mally et al. 1997). Increasing glutamine has been shown to protect cells against Aβ toxicity and provide neuroprotection in AD mice (Chen and Herrup 2012). Data obtained here showed different effects dependent upon protein, with increased glutamine levels upon challenge with Aβ40 fibrils, whereas a decrease was observed for Aβ42 oligomers and asyn fibrils (Fig. 5).

A side product of the glutamate/GABA-glutamine cycles is ammonia production (Fig. 6), elevated ammonia can alter a range of cellular functions and cause neurological symptoms including impaired memory (Bosoi and Rose 2009). Glutamate synthetase (GS) catalyses the reaction between ammonia and glutamate in the presence of adenosine triphosphate (ATP) to produce glutamine and adenosine diphosphate (ADP) (Fig. 6). ATP is largely increased upon addition of all proteins (Fig. 4), especially upon the addition of Aβ oligomers and asyn fibrils (Fig. 5).

l-Alloisoleucine is a branched chain amino acid present in human plasma and cerebrospinal fluid (CSF), accumulation of branched-chain compounds in blood and other body fluids has been suggested to have neurotoxic effects. We show a decrease in alloisoleucine when cells were challenged with predominantly monomeric proteins (Fig. 4).

### Neurotransmission

We observed alterations in several metabolites involved in neurotransmission (acetylcholine, glycine, taurine). Glycine acts as an inhibitory neurotransmitter in the central nervous system, and can employ glutamate as a co-agonist to facilitate an excitatory potential at the NMDA receptors. Higher glycine levels were observed in the CSF of AD patients (Jimenez-Jimenez et al. 1998) and plasma of PD patients compared to controls (Iwasaki et al. 1992). Here we show a variation in glycine levels dependent upon protein conformation (increase for monomer and oligomer, decrease in response to fibrils, Fig. 4).

In addition to the roles described above taurine can function as a neurotransmitter suggesting an alternative method by which taurine may be involved in disease progression. Glutamate is an excitatory neurotransmitter found to be significantly increased in the CSF of AD patients (Jimenez-Jimenez et al. 1998; Pomara et al. 1992), and decreased in the CSF of PD patients (Mally et al. 1997) suggesting that altered glutamate transmission may occur in these diseases. Enhanced glutamate levels reported in PD are thought to result as the body tries to compensate for a lack of dopamine, with glutamate antagonists proposed as therapeutics for PD (Blandini and Greenamyre 1998). Dopamine depletion also blocks autoinhibition of the neurotransmitter acetylcholine, leading to excessive acetylcholine release. In contrast, loss of cholinergic neurons in AD leads to a reduction in acetycholine and acetylcholine receptors (Kihara and Shimohama 2004; Pákáski and Kálmán 2008). Current AD treatment includes cholinesterase inhibitors (donepezil, rivastigmine and galantamine) which act to prevent acetylcholine degradation. This increase in acetylcholine can alleviate or stabilise AD symptoms. An additional treatment and NMDA receptor antagonist, memantine acts to block the effects of excess glutamate. We observed a slight increase in acetylcholine in the presence of Aβ fibrils and a decrease with asyn fibrils (Fig. 5). Pathway analysis suggested acetylcholine is involved in a range of different processes largely classified as affecting organismal systems (see Supplementary Table 3 in Supplementary Material). Disruption of the balance between excitatory (glutamate) and inhibitory (GABA/glycine) systems (as shown in Fig. 6) is thought to contribute to AD and PD pathogenesis, suggesting modulation as a therapeutic approach (Chumakov et al. 2015).

### Model limitations

Limitations of this study are that a cell-based assay cannot possibly reproduce all aspects of a complex organ such as the brain. Furthermore, neuroblastoma cells will not represent all processes observable in a non-immortalised cell line. Nevertheless, differences between treated and untreated cells are expected to report on pathways common to multiple neuronal cell types. The handling of cells post treatment will affect the metabolome, with trypsinisation thought to cause leakage of metabolites (Bi et al. 2013). However, trypsinisation was selected here due to the heterogeneity in cell number harvested by other methods (such as cell scraping). Thus although some additional cell permeability is expected the use of appropriate control groups should remove this effect from the relative metabolite changes observed. Finally this study identified three molecules (PEG, acetamide, ethanol) as varying with protein challenge that were removed from analysis as they were not related to neuronal cell metabolism. These molecules are not native to the cells and as such are likely to be introduced from the cellular environment (media). It is thought that the variability of their intracellular levels may be due to the increased membrane-permeability of cells exposed to amyloid proteins (Kayed et al. 2004) and as such report indirectly on the health of the cells and not relevant pathways for further analysis.

## Discussion

There have been a number of metabolomics studies investigating neurodegenerative diseases in animals and humans using tissue, blood, CSF or urine (Gonzalez-Riano et al. 2016). While these studies provide a wealth of information about cellular metabolism during disease and provide candidates for biomarker development using CSF (Andersen et al. 2017) or urine (Luan et al. 2015), it is difficult to isolate the effects due to amyloid proteins, specifically different protein conformers. Animal models of AD and PD have been employed in metabolomics studies (Kim et al. 2014a), however with new initiatives to reduce the number of animals used in research (e.g. NC3Rs) there is current interest to generate model systems which can investigate and manipulate cellular processes without the need for animals. Here we present a method using an intracellular neuronal metabolite profile to establish cellular processes directly affected by the amyloid aggregation process. It is anticipated that this approach that can be tailored to multiple specialised neuronal cell types will provide complementary information to studies carried out using tissue, CSF, urine and blood and thus help to understand the complex mechanisms and effects of different amyloidogenic protein conformers. This study has developed a robust method for monitoring metabolite profiles in a common neuronal cell line SH-SY5Y derived from neuroblastoma cells, as such the ability of these cells to reflect metabolite levels in in vivo neuronal cells is limited. However, the variance in metabolite levels when challenged with the various states of the amyloidogenic proteins are similar to metabolite changes noted in CSF studies suggesting that the model cell line does reflect aspects of the disease and is a valid substitute or additional tool to use alongside other human and animal models.

We have demonstrated that addition of fibrils produces metabolite profiles that are the most distinct from control cells (Fig. 2), with addition of predominantly monomers the most similar (Fig. 3). We determined the metabolites that contribute to the observed variance in profiles and analysed the effect of each protein and conformation on each of these metabolites in further detail (Figs. 4, 5). In general, oligomers and fibrils elicit different responses (Figs. 5, 6), suggesting that oligomers induce a stress response situation where cells try to compensate for the deleterious effects. However, when exposed to fibrils the data suggest that the system is overpowered causing dysregulation and cell damage, indicative of system shutdown (Cannino et al. 2012; Jha et al. 2012). This observation implies that fibrils may induce greater damage to cells than oligomers, consistent with DAPC analysis showing a greater effect on metabolite profiles in the presence of fibrils than observed for oligomers (Figs. 2, 3 and see Supplementary Fig. 5 in Supplementary Material).

Here this model has been probed for alterations to polar metabolites that reflect changes within cells following challenge with different conformations of proteins thought to represent the pathogenic process related to neurodegeneration. Further studies employing this cellular model with additional challenges, differentiated cell-types and deeper metabolite analysis via complementary techniques such as mass spectrometry will enhance the efficacy of this approach. However from this modest study many of the observed metabolite changes are consistent with previously reported observations in disease state through brain or CSF analysis we propose that the optimised workflow of methods and model presented here provide valuable insight into cellular processes and could be adapted for use in future drug screening in a regulated, controlled, calibrated, high throughput process in which many steps can be automated.

## Electronic supplementary material

Below is the link to the electronic supplementary material.

Supplementary material 1 (PDF 1237 KB)
