# Supplementary material for: Using an NMR metabolomics approach to investigate the pathogenicity of amyloid-beta and alpha-synuclein
Source: Metabolomics. 2017 Oct 29;13(12):151. doi: 10.1007/s11306-017-1289-5 (PMC5661010; doi:10.1007/s11306-017-1289-5)
Supplement: Supplementary file 1 — Supplementary material 1 (PDF 1237 KB) [file 11306_2017_1289_MOESM1_ESM.pdf]

Supplementary Information

Using an NMR metabolomics approach to investigate the pathogenicity of amyloid-beta and alpha-synuclein

Metabolomics

M.M. Phelan, E. Caamaño-Gutiérrez, M.S. Gant, R. Grosman, J. Madine
Institute of Integrative Biology, University of Liverpool, UK
Corresponding author contact: j.madine@liv.ac.uk

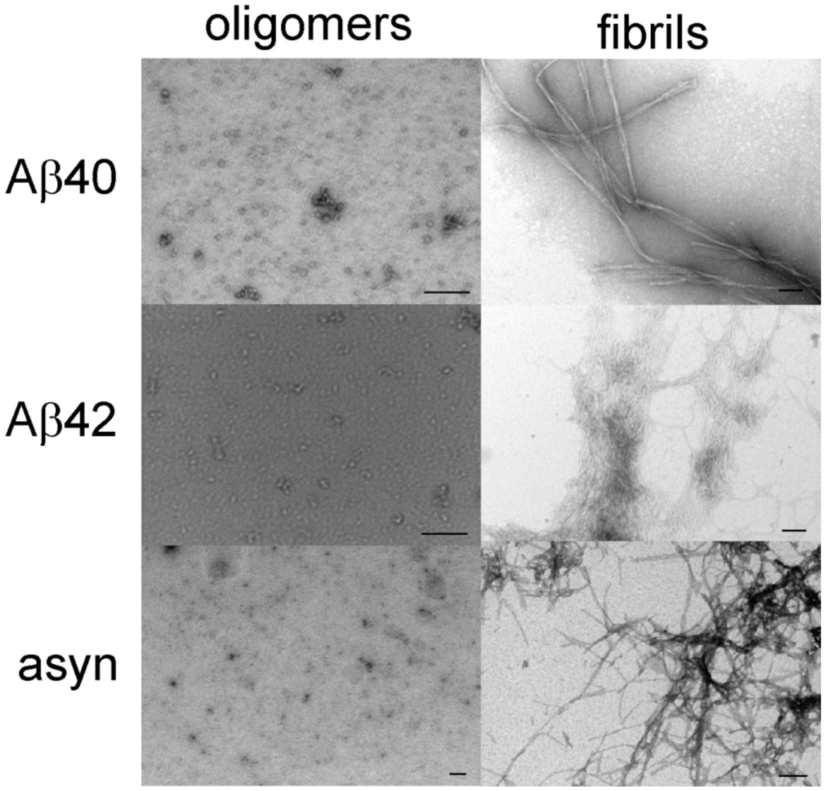

Supplementary Figure 1: Negative stain transmission electron microscopy for different forms of protein species (oligomers and fibrils) added to cells, scale bar is 200nm. Protein (5µl) was loaded onto carbon coated copper grids and negatively stained using 4% uranyl acetate, prior to visualisation on a Tecnai 10 electron microscope at 120 kV.

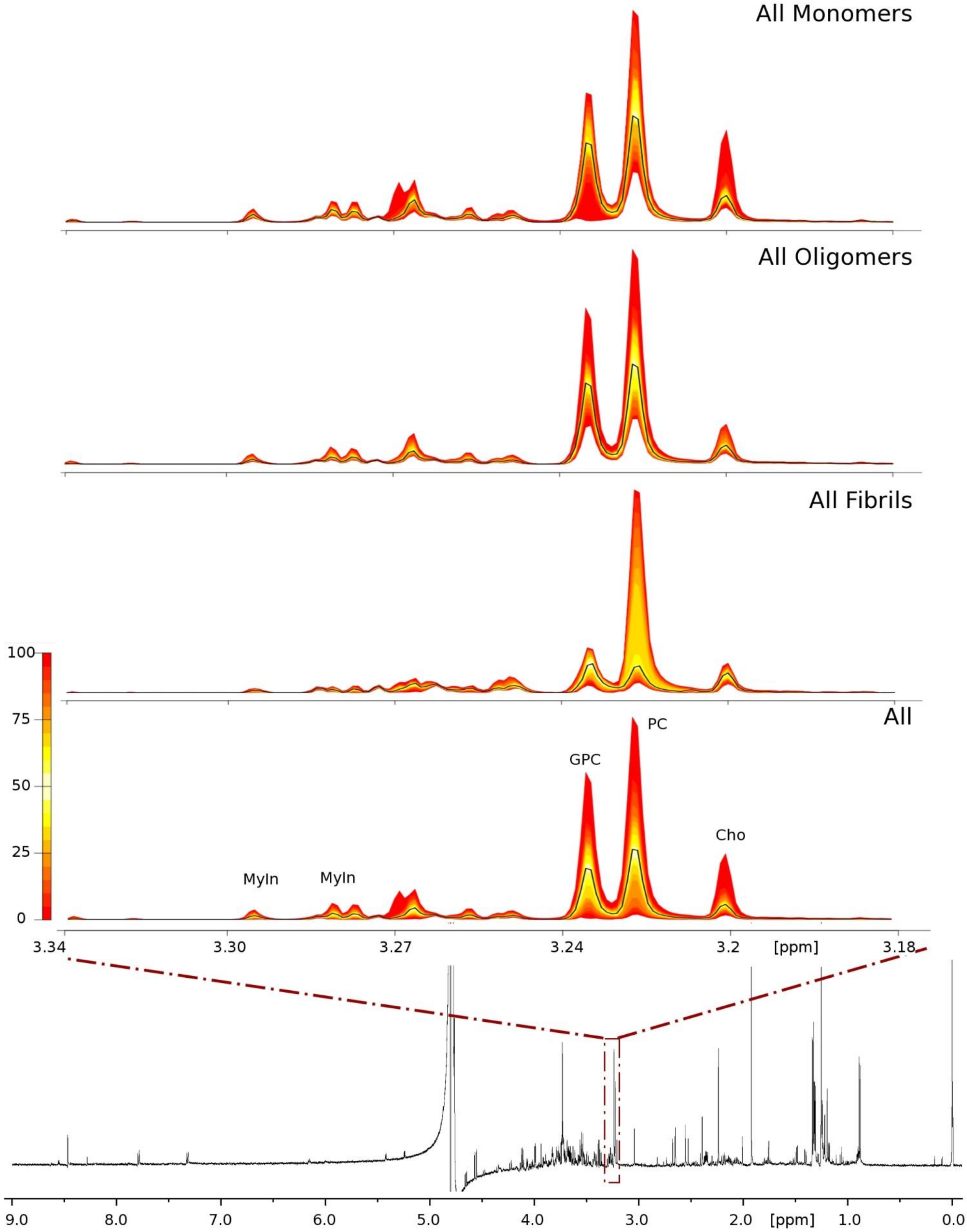

Supplementary Figure 2: Example ^{1}H NMR spectrum from the control group with quantile plot of spectra from neuroblastoma cells of boxed region (3.4-3.18ppm) expanded above, median spectra shown in black. All 33 spectra are shown in bottom 'All' quantile with key metabolites visible; choline (Cho), sn-glycerol-3-phosphocholine (3GPC), myoinositol (MyIn), phosphorylcholine (PC). Quantiles for monomer, oligomer and fibril challenges each include the respective nine sample groups.

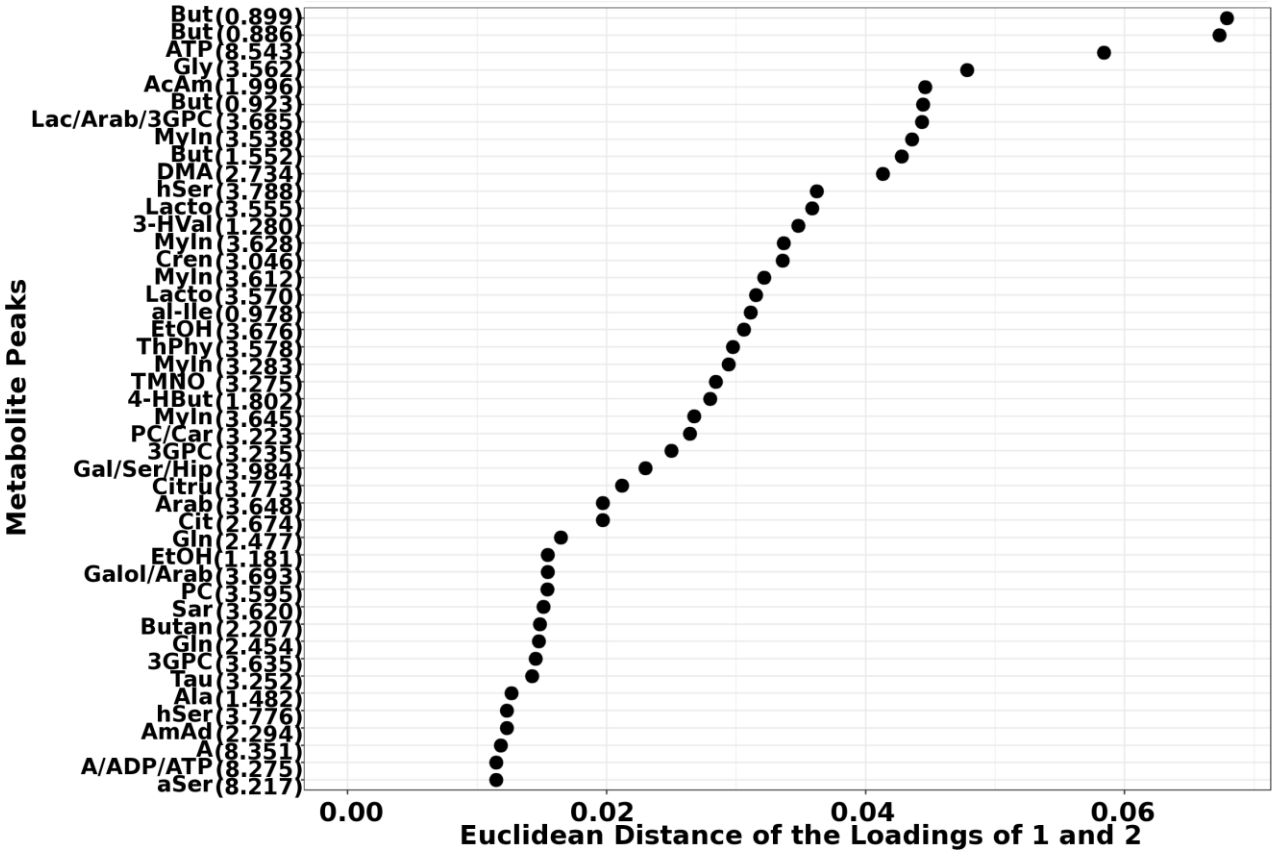

Supplementary Figure 3: Key metabolites that are identified to contribute to the top 25% variance between the groups for conformation analysis shown in Figure 2A with average ppm of peak indicated. Abbreviations used; Butyric acid (But), adenosine triphosphate (ATP), glycine (Gly), acetamide (AcAm), sn-glycero-3-phosphocholine (3GPC), myoinositol (MyIn), dimethylamine (DMA), homoserine (hSer), lactose (Lacto), 3-hydroxyvalerate (3-HVal), creatinine (Cre), alloisoleucine (all-Ile), ethanol (EtOH), theophylline (ThPhy), trimethylamine N-oxide (TMNO), 4-hydroxybutyrate (4-HBut), phosphorylcholine (PC), carnitine (Car), galactose (Gal), serine (Ser), hippurate (Hip), citrulline (Cit), arabinose (Arab), citrate (Cit), glutamine (Gln),galactinol (galol), sarcosine (Sar), butanone (Butan), taurine (Tau), alanine (Ala), 3-aminoadiapate (AmAd), adenosine (A), adenosine diphosphate (ADP), anserine (aSer).

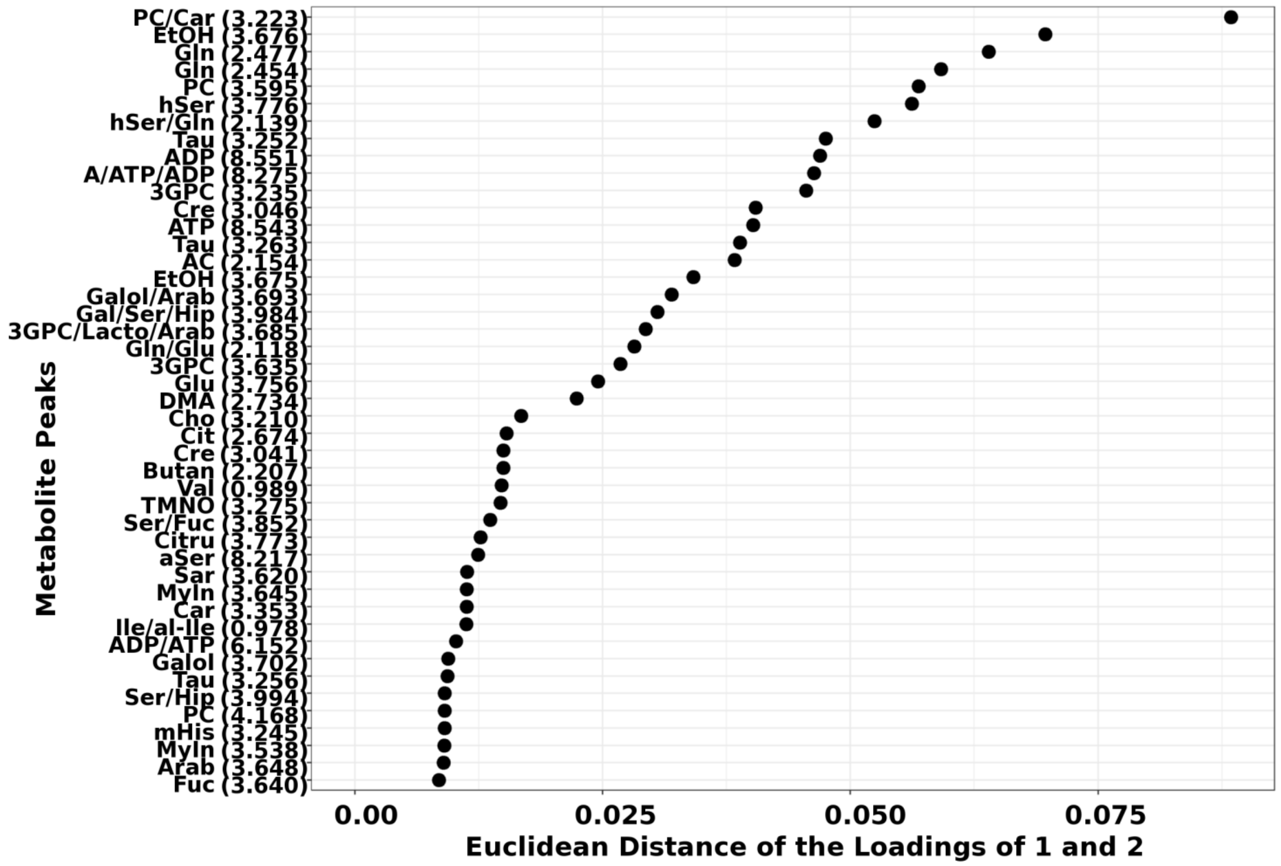

Supplementary Figure 4: Key metabolites that are identified to contribute to the top 25% variance between the groups for combined analysis shown in Figure 3A with average ppm of peak indicated. Abbreviations used; polyethylene glycol (PEG), acetylcholine (o-Ac), choline (Cho), valine (Val), fucose (FuC), isoleucine (Ile), pi-methyl-histidine (mHis).

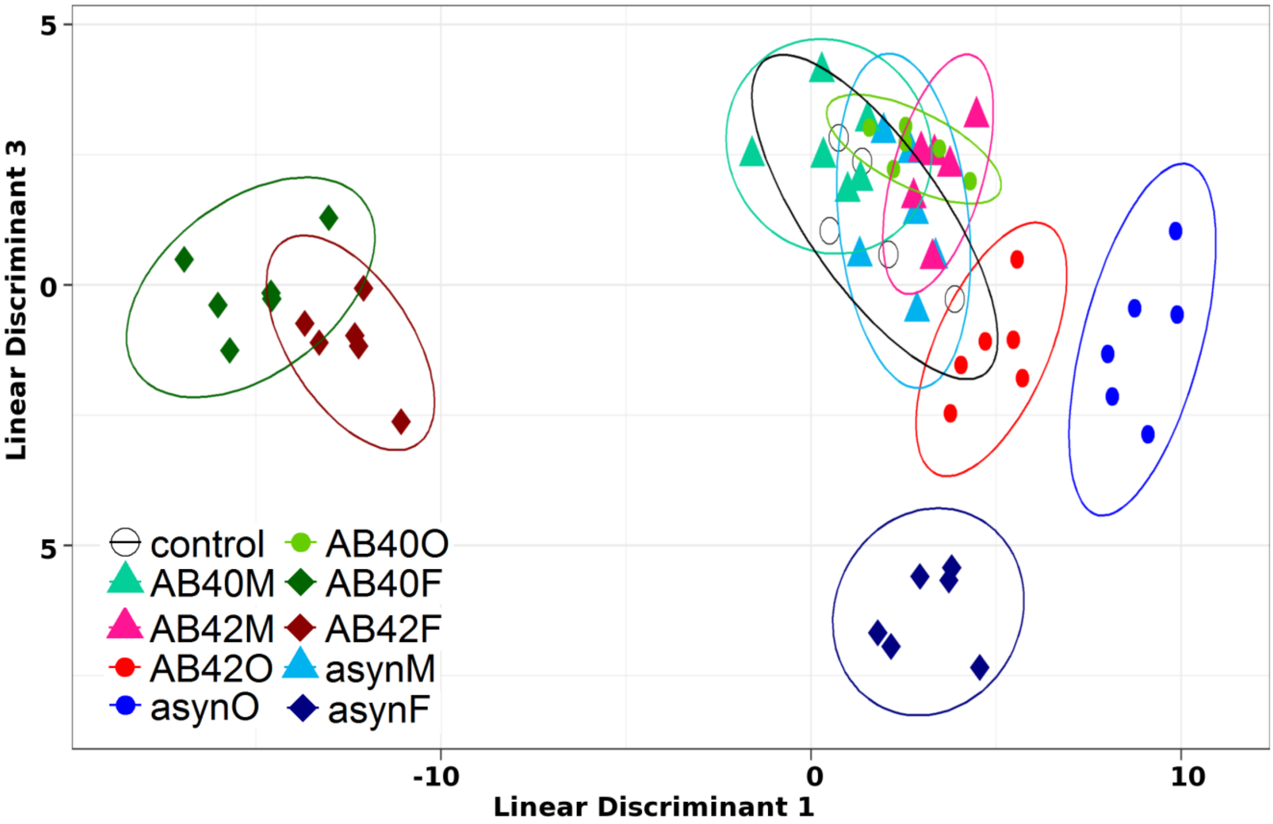

Supplementary Figure 5: Conformation DAPC model for linear discriminant 1 and 3. A) Separation of protein challenges; control (white circles), monomer (M), oligomer (O) and fibrillar (F) challenge for Aβ40 (green shades), Aβ42 (red shades) and asyn (blue shades). Greater separation is observed for asyn fibrils than shown in Figure 3A.

Supplementary Table 1: Metabolites identified to contribute to DAPC separation observed by ^{1}H NMR. Metabolite annotation was checked by correlation (for metabolites generating multiple distinct peaks). Metabolites with good covariance scores (> 0.5), with distinct peaks and thought to be relevant to neuronal cell metabolism are highlighted. Others were removed from biological contexualisation as described in the main text. The human metabolome database (HMDB) IDs for identified metabolites are given. The MSI assignment level is indicated according to recommendations by the Metabolomics Society Initiative (Salek et al. 2013).

		HMDB ID	Peaks	Overlap	Distinct	Correlation	MSI Identification level	Conformation top 10%	Combined top 10%
**Acetamide**	AcAm	31645	1	0	1	n/a	1	X	
**Acetylcholine**	AC	00895	1	0	1	n/a	1		X
**Adenosine**	A	00050	3	1	2	0.9	1		X
**Adenosine disphosphate**	ADP	01341	3	2	1	n/a	1		X
**Adenosine triphosphate**	ATP	00538	3	2	1	n/a	1	X	X
**L-alloisoleucine**	al-Ile	00557	3	2	1	n/a	2	X	
**Arabinose**	Arab	00646	12	6	6	(-0.7,0.9)	2		X
**butyric acid**	But	00039	4	0	4	(0.8,1.0)	1	X	
**Carnitine**	Car	2	1	3	0.0879	2	1		X
**Creatinine**	Cren	00562	1	0	1	n/a	2	X	X
**Dimethylamine**	DMA	00087	1	0	1	n/a	1	X	
**Ethanol**	EtOH	00108	5	1	4	(0.7, 1.0)	2		X
**(Poly)ethyleneglycol**	PEG	37790	1	0	1	n/a	1		X
**Galactitol**	Galol	00107	1	3	4	n/a	2		X
**Galactose**	Gal	00143	0	1	1	n/a	1		X
**L-glutamine**	Gln	00641	5	3	2	0.9	2		X
**Sn-glycero-3-phosphocholine**	3GPC	00086	13	6	7	(0.5,1.0)	1		X
**Glycine**	Gly	00123	1	0	1	n/a	2	X	
**Hippurate**	Hip	00714	1	2	3	n/a	1		X
**L-homoserine**	hSer	00719	7	2	5	(-0.8,0.6)	1	X	X
**Lactose**	Lacto	00186	15	6	9	(-0.6,0.9)	2	X	
**Myoinisitol**	MyIn	00211	6	0	6	(0.5, 1.0)	2	X	
**Phosphorylcholine**	PC	01565	3	1	2	1.0	1		X
**L-Serine**	Ser	00187	0	3	3	n/a	1		X
**Taurine**	Tau	00251	6	0	6	(0.7,1.0)	1		X
**3-Hydroxyvalerate**	3-HVal	00531	1	0	1	n/a	1	X	

Supplementary Table 2: DAPC models performance information.

MODEL	GROUP	PROPORTION OF OVERALL CORRECT ASSIGNMENT	PROPORTION OF CORRECT ASSIGNMENT PER GROUP
**CONFORMATION**	Control	93.22%	80.00%
	Monomers		83.33%
	Oligomers		100.00%
	Fibrils		100.00%
**COMBINED**	Control	89.83%	80.00%
	AB40M		83.33%
	AB40O		100.00%
	AB40F		100.00%
	AB42M		83.33%
	AB42O		100.00%
	AB42F		83.33%
	asynM		66.67%
	asynO		100.00%
	asynF		100.00%

Supplementary Table 3: Pathways associated with protein challenge models obtained from KEGG and MetaboAnalyst databases using significantly altered metabolites as input.

	CLASS	PATHWAY	ATP	Gly	Gln	Myln	Tau	But	3HVal	PC	3GPC	All-Ile	Cre	DMA	AC	HITS	TOTAL	%
CP	Cell motility	Regulation of actin cytoskeleton													X	1	6	16.7
	Trans. & catab.	Lysosome	X													1	4	25.0
EIP	Mem. Trans.	ABC transporters		X	X	X	X									4	126	3.2
	Sig. transduction	Calcium sig.	X													1	10	10.0
		cAMP sig.													X	1	40	2.5
		HIF-1 sig.	X													1	15	6.7
		Phosphatidylinositol sig. system				X										1	29	3.4
	Sig. molecules	Neuroactive ligand-receptor int.	X	X			X								X	4	130	3.1
GI	Translation	Aminoacyl-tRNA biosynthesis		X	X											2	52	3.8
Human Diseases	Cancers	Central carbon met. in cancer		X	X											2	37	5.4
		Choline met. in cancer								X	X					2	11	18.2
	Cardio dis.	Hypertrophic cardiomyopathy	X													1	5	20.0
	Endocrine & metabolic dis	Insulin secretion	X												X	2	13	15.4
		Type II diabetes mellitus	X													1	6	16.7
	Infect. dis	Pertussis	X													1	10	10.0
	Neuro. Dis	Parkinson's disease	X													1	15	6.7
		Nicotine addiction													X	1	7	14.3
Metabolism	Amino acid met.	Ala Asp & Glu met.			X											1	28	3.6
		Arg & Pro met.											X			1	76	1.3
		Arg biosynthesis			X											1	23	4.3
		Gly Ser & Thr met.		X												1	50	2.0
		Lys degradation		X												1	52	1.9
	Carbohydrate met.	Ascorbate & aldarate met.				X										1	47	2.1
		Butanoate met.						X								1	42	2.4
		Galactose met.				X										1	45	2.2
		Glyoxylate & dicarboxylate met.		X	X											2	61	3.3
		Inositol phosphate met.				X										1	44	2.3
	Energy met.	Nitrogen met.			X											1	19	5.3
		Oxidative phosphorylation	X													1	16	6.3
		Sulfur met.					X									1	32	3.1
	Lipid met.	Ether lipid met.									X					1	25	4.0
		Glycerophospholipid met.								X	X				X	3	52	5.8
		Primary bile acid biosynthesis		X			X									2	47	4.3
	Met. of cofactors & vit	Porphyrin met.		X												1	135	0.7
		Thiamine met.		X												1	31	3.2
		D-Glu & D-gln met.			X											1	12	8.3
		Glutathione met.		X												1	38	2.6
		Phosphonate & phosphinate met.		X												1	56	1.8
		Taurine & hypotaurine met.					X									1	22	4.5
	Nucleotide met.	Purine met.	X	X	X											3	92	3.3
		Pyrimidine met.			X											1	68	1.5
Organismal Systems	Digestive sys	Bile secretion													X	1	98	1.0
		Carbohydrate digestion & absorption						X								1	27	3.7
		Gastric acid secretion													X	1	14	7.1
		Mineral absorption		X	X											2	29	6.9
		Pancreatic secretion													X	1	17	5.9
		Protein digestion & absorption		X	X			X								3	47	6.4
		Salivary secretion													X	1	17	5.9
	Endocrine sys	Prolactin sig.	X													1	11	9.1
		Renin secretion	X													1	21	4.8
	Excretory sys	Prox tubule bicarb reclamation			X											1	17	5.9
	Immune sys	NOD-like receptor sig.	X													1	15	6.7
		Platelet activation	X													1	15	6.7
	Nervous sys	Cholinergic synapse													X	1	12	8.3
		GABAergic synapse			X											2	9	22.2
		Glutamatergic synapse			X											2	8	25.0
		Synaptic vesicle cycle	X	X											X	3	12	25.0
	Sensory sys	Inflam. regulation of TRP channels	X													1	36	2.8
		Taste transduction	X												X	2	32	6.3
		TOTAL METABOLITES	18 ATP	16 Gly	15 Gln	5 Myln	5 Tau	3 But	0 3HVal	2 PC	3 3GPC	0 All-Ile	1 Cre	0 DMA	13 AC			

Total column indicates total number of small molecule metabolites (compounds including transient intermediates and molecular entities not observable by NMR) reported in the pathway. Percentage indicates pathway coverage from metabolite shortlist shown. Abbreviations used; cardiovascular (cardio), catabolism (catab), cellular processes (CP), disease (dis), environmental information processing (EIP), genetic information processing (GI), infectious (infect), inflammatory (inflamm), interactions (int), membrane (mem), metabolism (met), neurodegenerative (neuro), signaling (sig), systems (sys), transporters (trans), vitamin (vit).

Supplementary Table 4: Metabolite set enrichment analysis (MSEA). Enrichment analysis using 26 metabolites identified in Supplementary Table 1. All significant pathways reported with P-values adjusted by Benjamini-Hochberg.

**Pathway**	**Pathway KEGG ID**	**Confidence Interval**	**Adjusted Pval**
ABC transporters - Homo sapiens (human)	hsa02010	(4.2656,35.3397)	0.001627
Neuroactive ligand-receptor interaction - Homo sapiens (human)	hsa04080	(2.9405,28.5527)	0.011506
Purine metabolism - Homo sapiens (human)	hsa00230	(2.7016,33.632)	0.021424
Galactose metabolism - Homo sapiens (human)	hsa00052	(3.0147,57.2774)	0.021669
Protein digestion and absorption - Homo sapiens (human)	hsa04974	(2.8898,54.5286)	0.021669
Synaptic vesicle cycle - Homo sapiens (human)	hsa04721	(4.0275,191.5)	0.022163
Parkinson's disease - Homo sapiens (human)	hsa05012	(3.2874,146.8167)	0.028152
Renin secretion - Homo sapiens (human)	hsa04924	(2.4029,100.7812)	0.045061

Supplementary Reference

Salek, R. M., C. Steinbeck, M. R. Viant, R. Goodacre, W. B. Dunn (2013). The role of reporting standards for metabolite annotation and identification in metabolomic studies. *GigaScience* 2, 13-13
